# pH-Stat Titration: A Rapid Assay for Enzymatic Degradability of Bio-Based Polymers

**DOI:** 10.3390/polym13060860

**Published:** 2021-03-11

**Authors:** Lukas Miksch, Lars Gutow, Reinhard Saborowski

**Affiliations:** Helmholtz Centre for Polar and Marine Research, Alfred Wegener Institute, Am Handelshafen 12, 27570 Bremerhaven, Germany; Lars.Gutow@awi.de (L.G.); Reinhard.Saborowski@awi.de (R.S.)

**Keywords:** polymer degradation, microparticles, PLA, PBS, enzymes, specificity, thermal profile, activation energy

## Abstract

Bio-based polymers have been suggested as one possible opportunity to counteract the progressive accumulation of microplastics in the environments. The gradual substitution of conventional plastics by bio-based polymers bears a variety of novel materials. The application of bioplastics is determined by their stability and bio-degradability, respectively. With the increasing implementation of bio-based plastics, there is also a demand for rapid and non-elaborate methods to determine their bio-degradability. Here, we propose an improved pH Stat titration assay optimized for bio-based polymers under environmental conditions and controlled temperature. Exemplarily, suspensions of poly(lactic acid) (PLA) and poly(butylene succinate) (PBS) microparticles were incubated with proteolytic and lipolytic enzymes. The rate of hydrolysis, as determined by counter-titration with a diluted base (NaOH), was recorded for two hours. PLA was hydrolyzed by proteolytic enzymes but not by lipase. PBS, in contrast, showed higher hydrolysis rates with lipase than with proteases. The thermal profile of PLA hydrolysis by protease showed an exponential increase from 4 to 30 °C with a temperature quotient Q_10_ of 5.6. The activation energy was 110 kJ·mol^−1^. pH-Stat titration proved to be a rapid, sensitive, and reliable procedure supplementing established methods of determining the bio-degradability of polymers under environmental conditions.

## 1. Introduction

As an attempt to substitute petroleum-based plastics, policy makers and producers are increasingly promoting the innovation and development of greener so-called “bioplastics” [1]. The term bioplastic is used for plastic material that is either bio-based, biodegradable, or both. The implementation of bio-based polymers as basis for the production of plastic goods is a promising alternative, as common reduce, reuse, and recycle strategies are insufficient to counteract the extensive accumulation of plastic waste in the environment [2]. With the perspective of having sustainably produced, biocompatible, and biodegradable materials, the development of bioplastics especially from waste and renewable resources has become popular [3]. Advances in waste reutilization [4,5] and in the synthesis technology of biopolymers make it possible to convert agricultural and food waste into usable bio-based materials. Natural polymers like poly(lactic acid) (PLA), polyhydroxyalkanoate (PHA), and polyhydroxybutyrate (PHB) have been isolated through different processes from by-products of milk, sugar, biodiesel, and other industrial processes [3,6]. The effective utilization of waste flows, low carbon footprint, and the biodegradability make bioplastics a sustainable option to replace conventional plastics.

Enzymatic biodegradation of synthetic polymers is, basically, a similar process as the degradation of natural polymers, such as cellulose or chitin [7]. Extracellular microbial enzymes attach to the surface of the polymer and form enzyme–substrate complexes. Accordingly, degradation rates are highest in irregular micro-particles with a high surface:volume ratio. These evolve in the environment through fragmentation of larger items by physicochemical and mechanical forcing. Depending on the substrate, specific enzymes hydrolyze the polymer chain and release oligo- and monomers. These degradation products may be finally assimilated and metabolized to water and carbon dioxide [8]. The enzymatic degradation is strongly influenced by environmental factors, such as pH and temperature [9,10], but also by the nature of the polymer [11,12]. Two common biodegradable polymers in commercial use are poly(lactic acid) (PLA) and poly(butylene succinate) (PBS). Both polymers are aliphatic polyesters and exhibit advantageous physical properties resembling those of polypropylene (PP) or low-density polyethylene (LDPE). Their chemical structure consists of monomers linked by ester bonds. Different hydrolytic enzymes, particularly esterases, are able to cleave these linkages. PLA was hydrolyzed in-vitro by serine proteases like proteinase K and subtilisin [13], whereas PBS was primarily hydrolyzed by lipase and cutinase [14,15].

Various standard tests and methods are available for the analytical assessment of the biodegradability of bioplastics [16]. These comprise the carbon dioxide forming test (Organisation for Economic Co-operation and Development, OECD 301b), differential scanning calorimetry (International Organization for Standardization, ISO 11357), weight-loss measurements (ISO 13432), or Fourier transform infrared spectroscopy [17]. Most of these methods are time consuming, laborious, or depend on expensive equipment. Accordingly, there is a demand for effective and accurate procedures that allow for the rapid analysis of the biodegradability of bioplastics under controlled conditions. A promising method is pH-Stat titration. It is based on maintaining a constant pH by the controlled addition of a diluted acid or base in a system where a pH affecting reaction takes place [18]. The hydrolysis of polyesters forms carboxyl groups, which reduce the pH in the solution. The quantity of added base to maintain a constant pH is thus a direct measure of the rate of hydrolysis of the bioplastic and, therefore, of its biodegradability. Although there is no uniform approach described, pH Stat titration has been used in several studies to assay the enzymatic degradation of aliphatic polyesters [19,20,21,22,23,24]. Most of these studies were conducted under elevated temperatures near enzyme optimum (Table 1). As the oceans are a potential sink of bio-based plastics, it is also of high relevance how natural environmental factors affect the process of degradation. Therefore, investigations of the hydrolysis of biopolymers under realistic environmental conditions are required.

In this study, we adapt the pH-Stat titration method for routine analysis to determine the biodegradability of bio-based plastics under relevant environmental conditions with support of specific proteolytic and lipolytic enzymes. Parameters for an improved performance and reliability of the method were tested and procedural difficulties identified.

## 2. Materials and Methods

### 2.1. Chemicals

Proteinase K from *Tritirachium album* (19133) was purchased from Qiagen (Hilden Germany) and the NaOH-standard solution (5564732) from Omnilab (Bremen, Germany). All other chemicals and enzymes were purchased from Sigma-Aldrich (St. Louis, MO, USA): poly(L-lactide) (PLA, 765112), poly(1,4-butylene succinate) (PBS, 448028), protease from *Bacillus licheniformis* (P4860), and lipase from *Candida antarctica* (62288) (Supporting Information, Supplementary Tables S1 and S2).

### 2.2. Titration Device

We used the automatic titrator TitroLine^®^ 7000 (SI Analytics GmbH, Mainz, Germany) for all pH-Stat applications. The titration unit was equipped with a 20-mL exchangeable head, a magnetic stirrer (TM 235), and a pH-electrode model A 162 2M DIN ID. The reaction vial was a 20-mL glass vial. The opening of the vial (17 mm) was too narrow to insert the electrode and the titration tip. Therefore, the titration tip was equipped with a 1 mm diameter polytetrafluoroethylene (PTFE)-tube. The reaction vial was placed in a custom-made thermostat jacket (Supporting Information, Figure S1). A circulation thermostat (e.g., Lauda, Lauda-Königshofen, Germany) connected to the thermostat jacket maintained a constant temperature in the reaction vial.

### 2.3. pH-Stat Titration

The titration system was set by default on the following parameters: mode: pH-Stat; endpoint: 8.2; step size: 0.001; measuring interval: 1 min; total time: 150 min; dosing speed: 1.5%; stirring speed: 1200 rpm. The pH was kept constant at 8.2 by titration of NaOH-solution. The concentrations ranged from 5 to 10 mmol·L^−1^, depending on the velocity of the reaction after the enzyme was added. Substrate (biopolymer) suspensions (3 mg·mL^−1^) were prepared in a solution of 3.4% sodium chloride in water (referred to as artificial seawater). PLA suspensions were prepared with a commercial product with an average M_n_ of 10,000. Aggregates of polymer particles were thoroughly crushed with a spatula before the suspension was sonicated in an ultrasonic bath for 8 min. Preliminary experiments showed that 3 mg·mL^−1^ was the highest concentration to ensure homogenous suspension of the particles and, thus, highest saturation. PBS granules were ground with a cryogenic mill (SPEX SamplePrep, 6775 Freezer/Mill) and sieved for fractions smaller than 200 µm. The suspensions were stirred in a glass beaker at 800 rpm for 16 h before aliquots of 10 mL were subjected to pH-Stat titration. Enzyme solutions (10–80 µL) were added to the reaction vial with a 100-µL microsyringe (Model 710 N, Hamilton Bonaduz AG, Bonaduz, Switzerland). The increase of volume due to titrant supply was always less than 1 mL. The electrode was calibrated every day before use. Routine measurements was carried out in triplicate.

### 2.4. Operational Parameters

To strengthen the quality standards, we separated the pH-Stat titration procedure into specific phases and separated unspecific background reactions from the true hydrolysis of the bioplastic material. To determine the optimal duration of the measurement, incubation experiments with PLA and protease from *Bacillus licheniformis* were run for up to 120 min. A linear regression was fitted to describe the amount of titrant (NaOH) added as a function of time. The coefficient of determination (R^2^) of the NaOH consumption curve was progressively calculated from the start to identify the period after which the coefficient of determination of the linear regression was consistently above R^2^ = 0.99.

### 2.5. Enzyme Specificity and Enzyme Concentration

Hydrolysis rates of PLA and PBS were determined with protease from *Bacillus licheniformis*, Proteinase K from *Tritirachium album*, and lipase from *Candida antarctica*. The hydrolysis rates were analyzed separately for PLA and PBS. Additionally, hydrolysis of PLA was assayed at five different protease concentrations (three replicates each) of up to 80 µL, which equals 30 to 240 mAU (Anson Units). Additionally, the dependency of the hydrolysis rate on the enzyme concentration was described by the following non-linear regression model:

(1) $$f_{\left(x\right)}\;=\;a+\left(b\;-\;a\right)\cdot \left(1\;-\;e^{\left(\;-\;c\cdot x\right)}\right)$$

with *a* being *f_(x)_* when *x* (concentration) is zero. *b* is the asymptote to which the curve approaches, which is the maximum reaction velocity. *c* is the rate constant of the curve.

### 2.6. Thermal Profiles

The temperature controlling system, consisting of a circulation cooler and a thermostat jacket, allowed maintaining constant and environmentally relevant temperatures during pH-Stat titration. Ten mL of a PLA-suspension (3 mg·mL^−1^) were incubated with 40 µL protease solution (120 mAU). The hydrolysis of PLA was measured at temperatures between 4 and 30 °C. The Q_10_ temperature coefficient was calculated as:

(2) $$Q_{10}={\left(\frac{V_{2}}{V_{1}}\right)}^{\frac{10}{T_{2}\;-\;T_{1}}}$$

where *V*_1_ and *V*_2_ represent the reaction velocities at the temperatures *T*_1_ and *T*_2_ in °C or K.

The apparent activation energy (*E_A_*) of the reaction was calculated from the slope of the Arrhenius plot:

(3) $$\ln V\;=\;-\;E_{A}\;\frac{1}{RT}\;$$

where *V* represents the reaction velocity, *R* is the universal gas constant, and *T* the temperature in K.

### 2.7. Statistics

For each of the two polymers (PLA and PHB), the hydrolysis rate was compared between the three enzymes (protease, Proteinase K, and lipase) by a 1-factorial analysis of variance (ANOVA) followed by Tukey’s HSD (honestly significant difference) test. Prior to the ANOVA, the data were tested for heteroscedasticity by Levene’s test. Similarly, the hydrolysis rates of PLA at five different protease quantities (30, 60, 120, 180, and 240 mAU) were compared by a 1-factorial ANOVA with subsequent Tukey’s HSD test for pairwise comparisons. The significance level of all statistical analyses was α = 0.05. Analyses and graphs were done with the program GraphPad Prism version 7.05 for Windows, GraphPad Software, La Jolla California USA, www.graphpad.com.

## 3. Results & Discussion

### 3.1. Duration and Sequence of pH-Stat Titration

Compared to natural substrates such as proteins, carbohydrates, or lipids, the enzymatic hydrolysis of bioplastics is slow. This is particularly due to the physical characteristics of the bioplastic particles. They are solid and do not dissolve in water. Only the surface of the particles is accessible to enzymatic action. Accordingly, the accurate determination of the in-vitro degradability of bioplastics requires a sensitive and reliable procedure.

**Figure 1:**
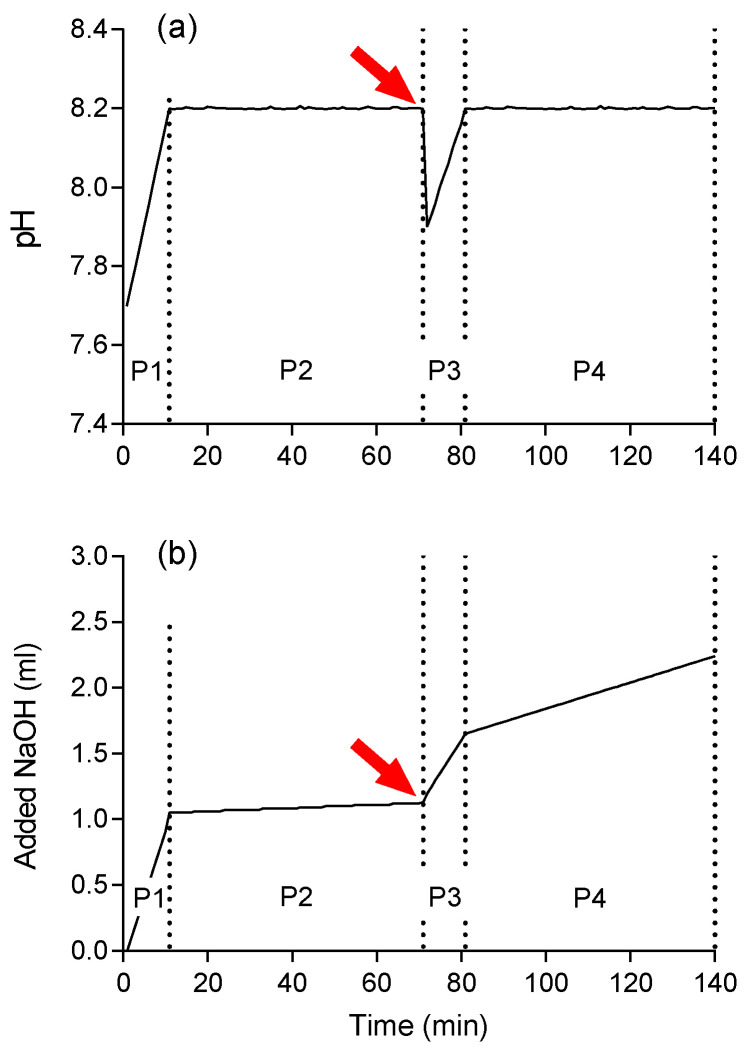
Typical course of (**a**) pH and (**b**) NaOH supply over time in a pH-Stat titration assay of a poly(lactic acid) (PLA) suspension as substrate and a PLA degrading enzyme. Phase 1 (P1): adjustment of the pH to 8.2. Phase 2 (P2): supply of low amounts of titrant to counterbalance an unspecific pH decrease before enzyme addition (substrate blank). Phase 3 (P3): re-adjustment of the pH to 8.2 after enzyme addition (indicated by an arrow). Phase 4 (P4): titration after enzyme addition to counterbalance a pH decrease of substrate hydrolysis (reaction) and an unspecific pH drop (blank).

The pH-Stat titration assay, as shown for the hydrolysis of PLA by a protease, follows a characteristic temporal profile of pH changes in the reaction vial and titrant (NaOH) supply, which can be separated into four distinct phases (Figure 1).

During Phase 1, the automatic titration system adjusts the pH of the suspension to the starting point of 8.2. Subsequently, the adjusted pH is monitored for 60 min (Phase 2) for conspicuous variations, which may reveal technical errors. During Phase 2, minor quantities of NaOH are continuously added because unspecific reactions in the suspension result in a slight decrease in pH (substrate blank). The addition of the slightly acidic enzyme solution induces a drastic pH drop in the suspension. After re-adjustment to pH 8.2 (Phase 3), NaOH is continuously added to counterbalance the decrease in pH in response to the formation of carboxyl groups from the enzymatic degradation of the substrate (Phase 4). Accordingly, the slope of the curve in Phase 4 is proportional to the hydrolysis rate plus the unspecific reactions of the substrate blank (Phase 2).

To evaluate the rates of unspecific reactions (substrate blank), we tested four substrate (PLA) concentrations (0, 1.5, 3, and 6 g·L^−1^) and five enzyme volumes of 10, 20, 40, 60, and 80 µL protease in 10 mL artificial seawater, corresponding to enzyme activities of 30, 60, 120, 180, and 240 mAU, respectively. The hydrolysis rate of the substrate blank (Phase 2) ranged between 8 and 26% of the enzyme-catalyzed reaction (Phase 4) for all enzyme concentrations. The blank reaction without enzyme did not correlate with the substrate concentration.

The unspecific reaction may be due to autolysis of biopolymers [25] or diffusion of atmospheric CO_2_ into the reaction mixture and dissociation to carbonic acid. We tried to minimize CO_2_ diffusion by overlaying the reaction mixture with an inert gas, such as nitrogen or helium. This, however, caused variations in temperature of the reaction solution and was not further considered. Another option to stabilize the initial pH is to use a buffer for suspension [22,23,24]. However, as the hydrolysis rates at environmental temperatures are expected to be rather low, a buffer could mask potential hydrolysis rates. Furthermore, a buffer does not represent natural conditions. Instead, we used seawater and corrected the slope of Phase 4 for the substrate blank (slope of Phase 2) to obtain the actual enzyme-catalyzed reaction V:

(4) $$V\;=\;c\;\cdot \left[\left(\frac{\delta v_{4}}{\delta t_{4}}\right)\;-\;\left(\frac{\delta v_{2}}{\delta t_{2}}\right)\right]\cdot 10^{6}$$

where *c* is the concentration of the added NaOH solution (mmol·L^−1^), *v*_2_ and *v*_4_ the volumes (mL) of NaOH added in phase 2 and 4, and *t*_2_ and *t*_4_ the duration (min) of the phases 2 and 4. The conversion factor 10^6^ was used to express the reaction velocity as nmol·min^−1^ under the given conditions. Additionally, the hydrolysis rate of the enzyme alone without substrate in artificial seawater (enzyme blank) was measured and subtracted from the hydrolysis rate of enzyme with substrate, to correct for possible autocatalytic activity of the enzyme solution.

### 3.2. Titrant Leakage

**Figure 2:**
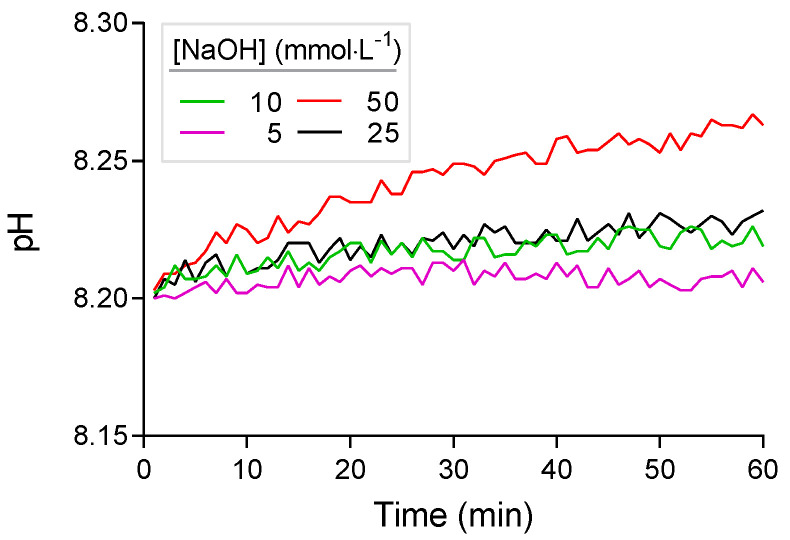
pH drift in artificial seawater over time with different concentrations of NaOH titrant due to diffusion from the titration tip.

After the initial adjustment of the pH in Phase 1, further increases in pH beyond the default value of 8.2 occurred during Phase 2 before the enzyme solution was added. This increase in pH was due to leakage of titrant from the tip of the titration device. To evaluate the magnitude of this leakage effect, the increase of the pH over time was analyzed by linear regression. The slope of the linear regression was tested for deviation from zero by an F-test. The increase in pH was more distinct when higher concentrated NaOH solutions were used (Figure 2). In artificial seawater, the increase was always statistically significant (i.e., slope of linear regression always significantly >0) and accounted for 0.01 pH units per hour with 5 mmol·L^−1^ NaOH and 0.02 to 0.06 pH units per hour at higher NaOH concentrations of 10 to 50 mmol·L^−1^. Upon request, the manufacturer of the titration device confirmed that small amounts of NaOH titrant may constantly diffuse from the titration tip into the reaction solution and raise the pH.

To minimize leakage, tips with different opening diameters were tested. However, NaOH diffused from the original tips of the manufacturer, as well as from custom-made tips. Similarly, reducing the opening diameter of the tip below 0.5 mm did not stop leakage of NaOH. To minimize this source of error, the NaOH titrant has to be adjusted to a concentration that does not significantly affect the measurement of the enzymatic activity. Simultaneously, the concentration of the NaOH has to be high enough to avoid excess addition of the titrant that may exceed the capacity of the reaction vial. The use of a buffer would also partially eliminate this increase in pH, but was not considered for reasons described above.

**Figure 3:**
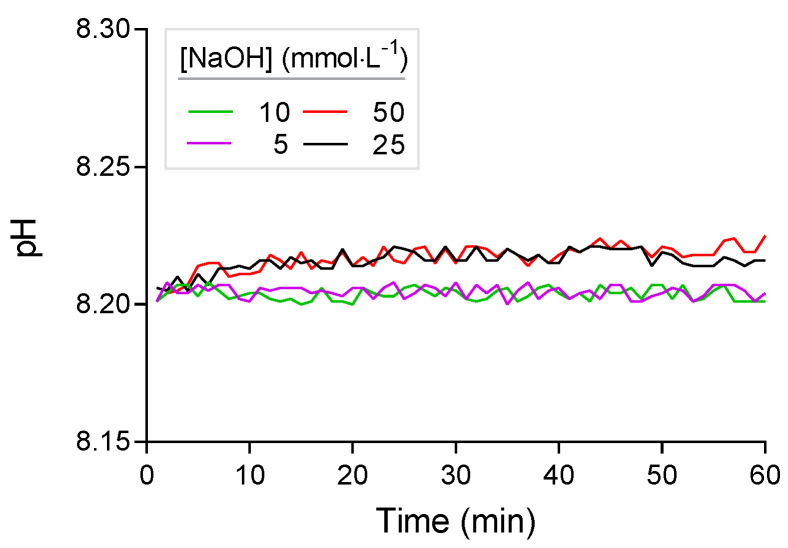
pH drift in substrate (PLA) suspension (3 mg·mL^−1^) in artificial seawater over time with different concentrations of NaOH titrant. Endpoint of the titration was set to 8.2. Titrant was added at NaOH concentrations of 5 and 10 mmol·L^−1^ (dashed lines) to counterbalance the pH drop (see main text).

When determining the leakage effect in PLA suspensions of 3 g·L^−1^, the increase of pH was lower than in artificial seawater (Figure 3). This is probably due to the unspecific acidifying reactions in the PLA suspension as observed in phase 2 of the titration profile (see Section 3.1). This unspecific reaction is stronger in PLA suspensions than in artificial seawater and therefore counterbalances the pH increase due to NaOH leakage. The pH increase in PLA-suspension of 3 mg·mL^−1^ was statistically significant for NaOH concentrations of 25 mmol·L^−1^ (F_1,58_ = 21.96, *p* < 0.01) and 50 mmol·L^−1^ (F_1,58_ = 66.27, *p* < 0.01). At lower NaOH concentrations of 10 and 5 mmol·L^−1^, the pH of the PLA-suspension dropped below 8.2 because of the unspecific reaction described above (3.1). Here, the titration system added small amounts of NaOH to keep the pH at 8.2. Therefore, NaOH concentrations of 10 mmol·L^−1^ (F_1,58_ = 0.01, *p* = 0.91) and 5 mmol·L^−1^ (F_1,58_ = 0.82, *p* = 0.37) showed no significant change of pH in the reaction vial.

### 3.3. Time Frame of pH-Stat Measurement

**Figure 4:**
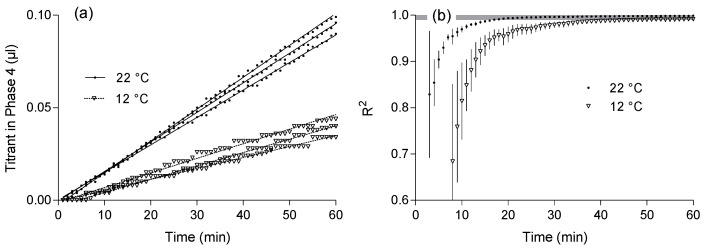
(**a**) Addition of titrant (0.01 mol·L^−1^ NaOH solution) during the 60 min lasting hydrolysis of PLA (3 g·L^−1^) by protease from *Bacillus licheniformis* (2.4 AU·mL^−1^). (**b**) Progressive approximation of the coefficient of variation to R^2^ = 0.99 (indicated by a grey bar).

With fast reactions due to high temperature (22 °C), the coefficient of determination reached R^2^ = 0.99 on average after 17 min in phase 4 of the pH-Stat titration assay. At lower temperatures (12 °C) and slower reactions, R^2^ = 0.99 was reached on average after 50 min (Figure 4).

Previous studies measuring hydrolysis rates at high temperatures had time frames ranging from 15 min (initial slope) up to several hours (Table 1). From the course and the coefficients of determination of the NaOH consumption curves (Figure 4) we concluded, that 20-min titration duration may be sufficient for fast reactions, whereas slow reactions showed strong pH variations after 30 min. Longer measurement periods of 60 to 120 min further increase the accuracy of the assay. However, depending on the enzyme used and the experimental conditions (temperature, pH) extended exposure times may be disadvantageous for less stable enzymes.

Therefore, prior testing of the enzyme activities under the specific assay conditions is recommended for measurements over longer time periods. Additionally, substrate limitation as a result of continuous substrate conversion will lead to a decrease in hydrolysis rate over time. Therefore, considering time constraints, throughput efficiency, and enzyme stability, we chose 60 min as an appropriate period for the pH-Stat assay of PLA hydrolysis in our study.

### 3.4. Effect of Enzyme Concentration

**Figure 5:**
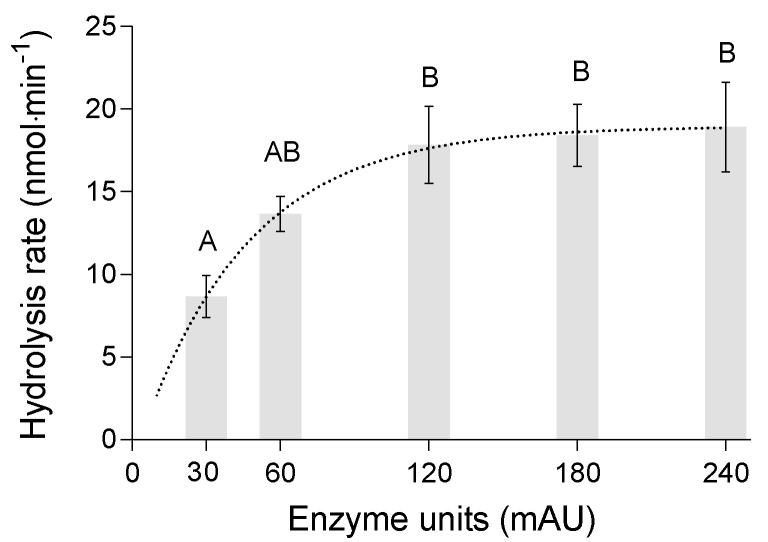
Rate of PLA hydrolysis (3 mg·mL^−1^) at different quantities (Anson Units) of protease from *Bacillus licheniformis* at 22 °C and pH 8.2 (means ± SD, *n* = 2–3).

The hydrolysis rate varied significantly with the amount of added enzyme (ANOVA: F_4,9_ = 12.71, *p* < 0.01–Figure 5). The average reaction velocity continuously increased from 8.0 ± 0.5 nmol·min^−1^ at 30 mAU to 17.9 ± 1.4 nmol·min^−1^ at 120 mAU. No statistically significant increase in reaction velocity was observed at volumes above 120 mAU. The non-linear regression model explained 85% of the variation and identified the maximum reaction velocity at 18.9 nmol·min^−1^. The calculated maximum velocity is within the range of the 95% confidence intervals of the reaction velocities at added enzyme amounts of 120–240 mAU.

The stagnation of the hydrolysis rate of PLA at higher volumes of the protease solution may be due to the limitation of accessible substrate at higher enzyme concentrations. The surface area of the polymer to which the enzymes can attach and hydrolyze the substrate is limited. If all accessible sites on the polymer are occupied, the cleavage and therefore the hydrolysis rate stagnates even if enzyme is added [26]. Similar results were reported for the hydrolysis of poly-(trimethylene succinate) films by lipase from *Rhizopus delemar* in pH-Stat degradation experiments [19].

### 3.5. Enzyme Specificity

**Figure 6:**
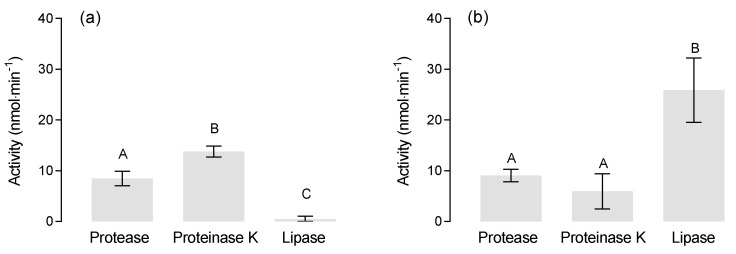
Enzymatic degradation of (**a**) PLA and (**b**) poly(butylene succinate) (PBS) at 22 °C and pH 8.2 (mean ± SD, *n* = 3). Different letters indicate statistically significant differences (Tukey multiple comparison test).

A set of enzymes was selected to examine their hydrolytic potential for PLA and PBS at 22 °C and pH 8.2. Both polymers were incubated with 100 µL lipase from *Candida antarctica* (18 U), 50 µL proteinase K from *Tritirachium album* (30 mAU), and 10 µL protease from *Bacillus licheniformis* (30 mAU), respectively. The hydrolytic activity for PLA varied significantly between the enzymes (ANOVA: F_(2,6)_ = 115.8, *p* < 0.0001–Figure 6a). PLA was efficiently degraded by the proteinase K from *Tritirachium album* with a hydrolysis rate of 13.7 ± 1.0 nmol·min^−1^ and by the protease from *Bacillus licheniformis* at 8.3 ± 1.0 nmol·min^−1^. The lipase from *Candida antarctica* showed almost no hydrolytic activity with 0.5 ± 0.6 nmol·min^−1^. Accordingly, both proteases were capable of degrading PLA, whereas the lipase was ineffective in PLA degradation. Efficient degradation of PLA by serine proteases has repeatedly been described [13,27,28]. However, the hydrolytic potential of most lipases is limited to aliphatic polyesters with low melting temperature and a lack of optically active carbon [29], which does not apply to PLA.

The hydrolytic activity for PBS also varied between the enzymes (ANOVA: F_(2,6)_ = 19.33, *p* = 0.0024–Figure 6b). Lipase from *C. antarctica* efficiently degraded PBS with a hydrolysis rate of 25.9 ± 5.3 nmol·min^−1^. The hydrolysis rates of protease from *B. licheniformis* and the proteinase from *T. album* were significantly lower at 9.1 ± 1.1 and 6.0 ± 1.2 nmol·min^−1^, respectively, although a potential of proteinase K for degrading PBS has been confirmed previously [22]. PBS is more efficiently hydrolyzed by lipases than proteases because of the conformational similarity of the polymer to tri- and diglycerides and the strong adsorption of lipase to the surface of PBS [30].

The degradation rates obtained by pH-Stat titration were verified with an alternative method. We chose a slightly modified fluorescent assay [31], which is based on the reaction of a fluorophore with liberated carboxyl groups of the PLA. The fluorescent method confirmed that protease from *B. licheniformis* and proteinase K from *T. album* were able to hydrolyze PLA (Supporting Information, Figure S2). However, the fluorescent assay is specific with regard to the polymer type and not applicable to measure the degradation products of PBS. It is also less sensitive than the pH-Stat method. Accordingly, the pH-Stat titration method is applicable to a wider range of polymers and performs better in in-vitro assays on small polymer quantities.

### 3.6. Thermal Profiles

Temperature controlled pH-Stat titration allows for investigating biodegradability of polymers within a wide range of environmentally relevant temperatures from 4 °C to above 30 °C. However, adjusting the temperature of the reaction mixture below 4 °C was challenging because of heat generation by the stirring unit, which is necessary to dispense the titrated NaOH in the reaction vial efficiently.

**Figure 7:**
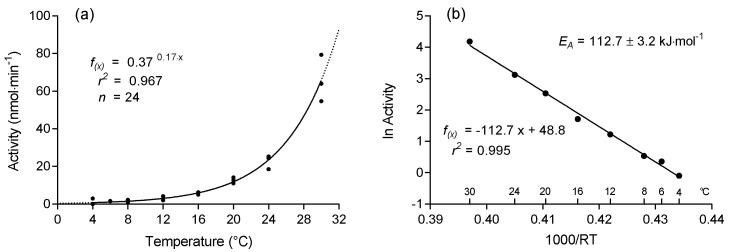
(**a**) Hydrolysis rates of PLA at different temperatures. (**b**) Arrhenius-plot calculated from data of the temperature profile of PLA hydrolysis.

The hydrolysis rate of PLA by *B. licheniformis* protease strongly increased with temperature and followed first-order kinetics (Figure 7a). Activities ranged from 1.4 ± 0.2 nmol·min^−1^ at 4 °C to 66.0 ± 10.2 nmol·min^−1^ at 30 °C. The temperature coefficient Q_10_ of 5.6 (calculated over the entire temperature range from 4 °C to 30 °C) reveals that a rise in temperature by 10 °C induces a 5.6-fold increase in hydrolytic activity.

Degradation of PLA by protease (*B. licheniformis*) proceeded more slowly at environmentally relevant seawater temperatures of up to 20 °C compared to seawater temperatures above 25 to 30 °C. Such elevated temperatures occur only in tropical waters [32,33] or in exposed rock pools warmed by solar radiation during low tide [34,35]. On the ground, highest temperatures appear at the surface and depend strongly on irradiation, soil humidity, and topography. At beaches, surface temperatures increase with distance from the shoreline and may exceed 50 °C on dry sand [36,37] but rapidly cool down already a few decimeters below the surface. Accordingly, temperature may become a limiting factor in the decomposition of biodegradable plastics in natural habitats [38]. Consequently, industrial composting processes typically utilize temperatures of 50 to 65 °C to accelerate the degradation process [16,39,40].

The Arrhenius-plot showed a strong linear relation between the ln of the reaction velocity and the reciprocal of the absolute temperature (Figure 7b). The apparent activation energy (*E_A_*) of the hydrolysis of PLA by *B. licheniformis* protease was 112.7 ± 3.2 kJ mol^−1^. The activation energy of a chemical reaction is a proxy for the velocity of the reaction. The lower the activation energy, the easier and faster a reaction can proceed.

Thermodynamic data on the hydrolysis of biodegradable polymers at environmentally relevant temperatures are lacking whereas data on thermal degradation at high temperatures of up to several hundred degree Celsius are available. For example, the activation energy of the thermogravimetric degradation of corn starch-based plastic accounts for 120–140 kJ·mol^−1^ [41]. The activation energy of the thermodegradation of hemp-poly lactic acid composites was about 160 kJ·mol^−1^ [42]. Those authors suggested that a high binding energy of the polymers improves the thermal stability of the compound, as it requires a higher activation energy to induce degradation.

Activation energies of thermogravimetric decomposition of natural substrates or their derivatives are in the same range. The E*_A_* of cellulose from different plants are between 150 and 200 kJ·mol^−1^ [43] whereas the apparent E*_A_* of the thermogravimetric decomposition of chitin and chitosan account for 125 and 169 kJ·mol^−1^, respectively [44].

The E*_A_* of enzymatic degradation of natural polymers is distinctly lower. Cellulolytic activity in soil showed activation energies of 22–28 kJ·mol^−1^ [45]. The E*_A_* of the degradation of cellulose films by cellulose from sac fungus *Trichoderma reesei* was 37 kJ·mol^−1^ [46]. Similarly, the hydrolysis of swollen chitin by chitinase from *Trichoderma harzianum* showed an E*_A_* of 70 and 80 kJ·mol^−1^ [47] and from *Paenibacillus* sp. of 19 kJ·mol^−1^ [48]. Chitinases of the psychrophilic Antarctic bacterium *Arthrobacter* sp. showed E*_A_* of 62–63 kJ·mol^−1^ when hydrolyzing soluble chitin [49].

The capability of our method to establish thermal profiles rapidly and to deduce thermodynamic parameters allows studying material characteristics for various purposes. These may comprise industrial or medical applications, degradation kinetics, and effects of weathering. These results may be important to evaluate the degradability of bio-based polymers under conditions of the natural environment as well as industrial composting facilities.

### 3.7. Limitations

Despite many advantages, the pH-Stat titration method as described here has also limitations.

(1) Buffering Capacity of Plastic Suspensions

The pH of a polymer suspension depends on the properties of the liquid (water) and the polymer used. This intrinsic pH of the suspension was usually around 8.0 in our experiments with seawater and PLA/PBS. In phase 1 of each titration assay, the pH of the suspension was adjusted to 8.2, at which the hydrolytic reaction was observed.

Seawater or freshwater has a negligible buffer capacity. This has the advantage that even the lowest hydrolysis rates are detectable. However, due to the negligible buffering capacity, we observed that the adjusted pH moved towards the original intrinsic pH of the suspension with time. This may be due to the interaction of the plastic particles or leaching additives with the water.

If the intrinsic pH is lower than the adjusted pH, a decrease in pH appears in phase 2 (substrate blank) and is compensated by the addition of sodium hydroxide. If the intrinsic pH is higher than the adjusted pH, no sodium hydroxide will be added when the pH is increasing and no net change will be measured. This may mask a potential reaction and lead to an underestimation of the hydrolysis rate. To ensure the most accurate measurement of the hydrolysis rate, the adjusted pH should always be slightly higher than the intrinsic pH of the polymer suspension.

(2) Particle Size and Surface Area

The enzymatic reaction takes place in a suspension of very small particles. These particles (powder) have to be prepared first by e.g., cryo-milling and sieving. The enzymes act at the surface of the particles, which, however, cannot be defined in small mg-amounts of sample. Surface characteristics may vary between plastic types.

## 4. Conclusions

pH-Stat titration is a valuable tool for investigating the biodegradability of polymers, which may ideally complement already existing testing methods or even substitute some of them. When screening bio-based and other polymers for their degradability by various enzymes, the pH-Stat titration provides rapid and reliable results within only a few hours instead of weeks or months. To ensure a smooth and accurate performance of this method under environmental conditions, the tuning of the parameters to the chosen conditions and selected test materials is necessary.

The precise measurement of pH and base consumption, which reflect the formation of carboxyl groups through hydrolysis of the polymer, allows for a high sensitivity of the method and dealing with extremely small polymer quantities. For PLA and PBS microplastics we were able to demonstrate differential enzymatic degradability by proteolytic enzymes and lipase, respectively, and to describe the temperature dependencies of hydrolysis rates. This method is also suitable to monitor the enzymatic biodegradability of any other biopolymer, including future inventions facing the challenge to reduce environmental pollution by persistent plastic materials. No buffer solutions or other chemical reagents, which may interfere with the hydrolytic reaction, are required in the reaction solution to keep the pH constant during the measurement. Accordingly, degradation processes in natural media, such as freshwater or seawater, can be studied accurately. This makes this method an excellent tool for studying polymer degradation under different environmentally relevant conditions in terms of temperature, salinity, or pH. Additionally, the resulting degradation products, i.e., oligomers and monomers, can be isolated and subjected to detailed chemical analysis for studying each step in the process of polymer decomposition, which is also beneficial in the development of innovative future materials.

## Figures and Tables

**Table 1:** Procedural parameters of reported polymer degradation experiments by pH Stat titration.

Polymer (Substrate)	Enzyme ^(Source)^	Temperature	Medium	Period	Reference
Poly-(trimethylene succinate)	Lipase ^a^	37 °C	0.9% NaCl	10 h	[19]
Several model polyesters	Lipase ^b,c,d,e,f^ Hydrolase ^g^ α-Chrymotrypsin ^h^ Subtilisin ^i^ Esterase ^j^	25–50 °C	0.9% NaCl	15 min–20 h	[20]
Several model polyesters	Lipase ^b,e,k,l,m,n,o^ Hydrolase ^p^ Proteinase K	40 °C	0.9% NaCl	15 min	[21]
Poly(3-hydroxybutyrate) (PHB)	PHB depolymerases	37 °C	1 mmol·L^−1^ Tris-HCl	20 min	[22]
Poly(ethylene terephtalate) (PET)	Cutinase ^q,r,s^	30–90 °C	1 mmol·L^−1^ Tris-HCl with 10% glycerol	15 min	[23]
Poly(vinyl acetate) (PVC)	Cutinase ^q,r,s^	40 °C, 50 °C, 70 °C	1 mmol·L^−1^ Tris-HCl with 10% glycerol	1–192 h	[24]

^a^ Rhizopus delemar, ^b^ Pseudomonas spp., ^c^ Chromobacterium viscosum, ^d^ Rhizomucor miehei, ^e^ Candida cylindracea, ^f^ wheat germ, ^g^ Thermonospora fusca, ^h^ bovine pancreas, ^i^ Bacillus subtilis, ^j^ porcine liver, ^k^ Aspergillus oryzae, ^l^ Mucor miehei, ^m^ porcine pancreas, ^n^ Pseudomonas cepacia, ^o^ Pseudomonas fluorescens, ^p^ Thermobifida fusca, ^q^ Humilica insolens, ^r^ Fusarium solani, ^s^ Pseudomonas mendocrina.

## Data Availability

The data presented in this study were submitted to PANGAEA to be openly available.

## Supplementary Materials

The following Supplementary Materials are available online at https://www.mdpi.com/2073-4360/13/6/860/s1, Figure S1: Technical illustration of the thermal jacket, Figure S2: Fluorometric assay of PLA degradation products, Table S1: Enzyme specifications, Table S2: Polymer specifications
