# Supplementary material for: pH-Stat Titration: A Rapid Assay for Enzymatic Degradability of Bio-Based Polymers
Source: Polymers (Basel). 2021 Mar 11;13(6):860. doi: 10.3390/polym13060860 (PMC7998482; doi:10.3390/polym13060860)
Supplement: Supplementary file 1 [file polymers-13-00860-s001.pdf]

polymers

MDPI

Supporting Information

pH-Stat Titration: A Rapid Assay for Enzymatic Degradability of Bio-based Polymers

Lukas Miksch [1,*], Lars Gutow [1] and Reinhard Saborowski [1]

[1] Alfred Wegener Institute, Helmholtz Centre for Polar and Marine Research, Am Handelshafen 12, 27570 Bremerhaven, Germany
* Correspondence: Lukas.Miksch@awi.de; ORCID: http://orcid.org/0000-0001-8236-2336; Tel.: +494714831-1326

Content:

1. Technical illustration of the thermal jacket

2. Fluorometric assay of PLA degradation products

3. Table S1: Enzyme specifications

4. Table S2: Polymer specifications

1. Thermal jacket

The reaction vial was a 20-mL glass vial with an opening of 17 mm. The vial was placed in a custom-made thermostat jacket (Figure S1). A circulation thermostat (e.g. Lauda, Lauda-Königshofen, Germany) connected to the thermostat jacket maintained a constant temperature in the reaction vial.

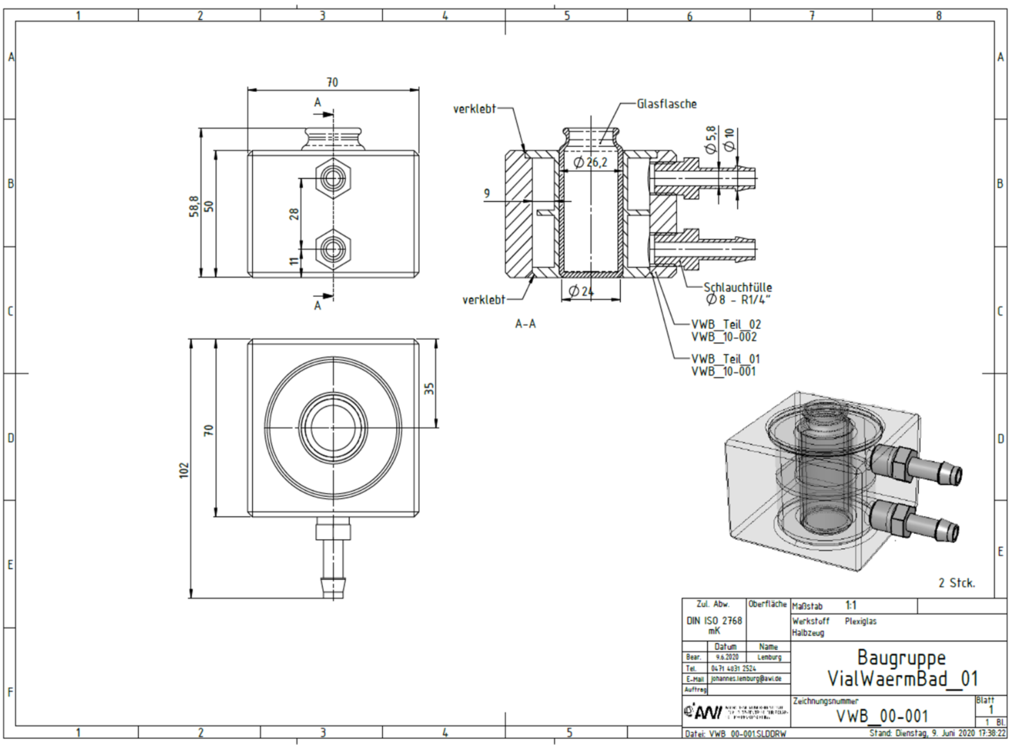

Figure S1. Technical illustration of the thermal jacket and the reaction vial used in pH-Stat titration.

2. Fluorometric Assay of PLA Degradation Products

The products of the enzymatic degradation of poly(L-lactide) were assayed after Vichaibun & Chulavatnatol [1] with modifications for the application in microplates. This fluorescent assay is based on the reaction of the carboxyl groups of lactic acid and its soluble polymers with the fluorescent agent o-phthalaldehyde (OPA) in the presence of 1-ethyl-3-(3-dimethylaminopropyl) carbodiimide (EDC) [2]. 15 mg of PLA suspended in 0.5 mL Sorensen's phosphate buffer (pH 8.2) were incubated with different amounts of enzyme solutions at 30 °C in a thermostatic block (Eppendorf, Thermomixer comfort). Samples of 50 µL were extracted from the reaction tube and immediately centrifuged at 20,000 g and 4 °C to sediment PLA particles. The supernatants were transferred into new reaction tubes. Subsequently, 25 µL of the supernatant were mixed with 25 µL of EDC solution (4 % w/v), which was freshly prepared in 0.1 mol·L-1 TEMED puffer, pH 4.75. The mixture was incubated for 30 minutes at 30 °C in a thermostatic block (Eppendorf, Thermomixer comfort). Thereafter, 20 µL of the mixture were mixed with 400 µL of OPA reagent and 800 µL of water and transferred into microplate wells. The fluorescence was measured in a Thermo Fisher Ascent FL microplate reader at λex = 340 nm and λem = 455 nm. Blanks without sample were run in parallel. A standard curve was prepared with different concentrations of lactic acid.

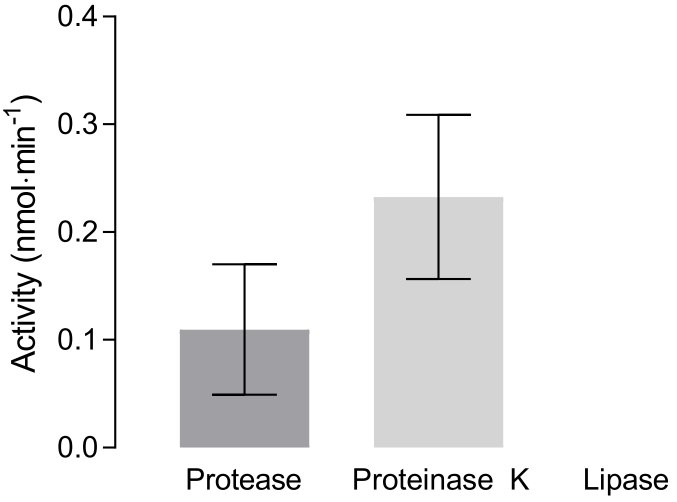

Figure S2. Fluorescence signal of the degradation products of PLA after 1 h incubation with protease, proteinase K and lipase at 30°C.

Incubation of PLA with proteinase K from *Tritirachium album* showed the highest activity liberating 0.23 ± 0.08 nmol·min-1 of lactic acid equivalents. The protease from *Bacillus licheniformis* showed lower activity of 0.12 ± 0.06 nmol·min-1 of lactic acid equivalents. No fluorescence was observed after incubation with lipase from *Candida antarctica*. The results obtained by pH Stat titration were much higher than that from the fluorescence assay. Although the fluorescence assay was performed at higher temperature and substrate concentration, it showed much lower sensitivity than the pH Stat titration assay. No reaction products were detected with the fluorescence assay after incubation of PBS with protease, proteinase K, and lipase. Therefore, pH Stat titration appears to be more versatile as in-vitro assay for bioplastic degradation than the fluorescence assay.

3. Table S1. Enzyme specifications

Enzyme	Source	Enzymatic activity	Distributor	Produt No.
Protease	*Bacillus licheniformis*	2.4 AU/g [a]	Sigma	P4860
Proteinase K	*Tritirachium album*	318 mAU/mL [b]	Sigma	19133
Lipase	*Candida antarctica*	9 U/mg [c]	Sigma	62288

[a] *One Unit will hydrolyze casein to produce color equivalent to 1.0 mmol (181 µg) of tyrosine per minute at pH 7.5 at 37 °C (color by Folin-Ciocalteu reagent).*

[b] *One mAU is the activity that releases folin-positive amino acids and peptides corresponding to 1 µmol tyrosine per minute.*

[c] *One Unit corresponds to the amount of enzyme which liberates 1.0 µmol butyric acid per minute at pH 8.0 and 50°C.*

4. Table S2. Polymer specifications

Polymer	Average M_n	Cristallinity (%)	T_m (°C)	Distributor	Product No.
Poly-(L-lactid)	10,000	70	162-167	Sigma	765112
Poly(1,4-butylene succinate), extended with 1,6-diisocyanatohexane	No data	No data	120	Sigma	448028

References

1. Vichaibun, V.; Chulavatnatol, M. A new assay for the enzymatic degradation of polylactic acid. *Sci. Asia* 2003, 29, 297-300; https://doi.org/10.2306/scienceasia1513-1874.2003.29.297.
2. Kobayashi, M.: Ichishima, E. Use of water-soluble 1-ethyl-3 (3-dimethylaminopropyl) carbodiimide for the fluorescent determination of uronic acids and carboxylic acids. *Anal. Biochem*. 1990, 189(1), 122-125; https://doi.org/10.1016/0003-2697(90)90056-F.
